# Comparison of Voxel-Wise Tumor Perfusion Changes Measured With Dynamic Contrast-Enhanced (DCE) MRI and Volumetric DCE CT in Patients With Metastatic Brain Cancer Treated with Radiosurgery

**DOI:** 10.18383/j.tom.2016.00178

**Published:** 2016-12

**Authors:** Catherine Coolens, Brandon Driscoll, Warren Foltz, Carly Pellow, Cynthia Menard, Caroline Chung

**Affiliations:** 1 Radiation Medicine Program, Princess Margaret Cancer Center and University Health Network, Toronto, Ontario, Canada;; 2 Department of Radiation Oncology, University of Toronto, Toronto, Ontario, Canada;; 3 Institute of Biomaterials and Biomedical Engineering, University of Toronto, Ontario, Canada;; 4 TECHNA Institute, University Health Network, Toronto, Ontario, Canada; and; 5 Department of Radiation Oncology, University of Montreal Hospital, Montreal, QC, Canada

**Keywords:** DCE MRI, tumor response, quantitative imaging, biomarker, CT perfusion

## Abstract

Dynamic contrast-enhanced (DCE)-MRI metrics are evaluated against volumetric DCE-CT quantitative parameters as a standard for tracer-kinetic validation using a common 4-dimensional temporal dynamic analysis platform in tumor perfusion measurements following stereotactic radiosurgery (SRS) for brain metastases. Patients treated with SRS as part of Research Ethics Board-approved clinical trials underwent volumetric DCE-CT and DCE-MRI at baseline, then at 7 and 21 days after SRS. Temporal dynamic analysis was used to create 3-dimensional pharmacokinetic parameter maps for both modalities. Individual vascular input functions were selected for DCE-CT and a population function was used for DCE-MRI. Semiquantitative and pharmacokinetic DCE parameters were assessed using a modified Tofts model within each tumor at every time point for both modalities for characterization of perfusion and capillary permeability, as well as their dependency on precontrast relaxation times (TRs), *T*_10_, and input function. Direct voxel-to-voxel Pearson analysis showed statistically significant correlations between CT and magnetic resonance which peaked at day 7 for K_trans_ (R = 0.74, *P* ≤ .0001). The strongest correlation to DCE-CT measurements was found with DCE-MRI analysis using voxel-wise *T*_10_ maps (R = 0.575, *P* < .001) instead of assigning a fixed *T*_10_ value. Comparison of histogram features showed statistically significant correlations between modalities over all tumors for median K_trans_ (R = 0.42, *P* = .01), median area under the enhancement curve (iAUC_90_) (R = 0.55, *P* < .01), and median iAUC_90_ skewness (R = 0.34, *P* = .03). Statistically significant, strong correlations were found for voxel-wise K_trans_, iAUC_90_, and v_e_ values between DCE-CT and DCE-MRI. For DCE-MRI, the implementation of voxel-wise *T*_10_ maps plays a key role in ensuring the accuracy of heterogeneous pharmacokinetic maps.

## Introduction

Dynamic contrast-enhanced (DCE) imaging can be useful for evaluating vascular injury and endothelial permeability changes following radiation therapy, including ablative therapy such as stereotactic radiosurgery (SRS) or when combined with antiangiogenic therapy (1). Preclinical work in a murine intracranial glioma model showed that early quantitative changes in diffusion and perfusion magnetic resonance imaging (MRI) metrics reflect treatment responses soon after initiating combinatorial radiation and antiangiogenic therapies (2).

The development of DCE-MRI techniques has seen a rapid growth in its translation into the field of radiation therapy clinical trials (3, 4), but DCE-MRI measures of tumor vascular physiology have shown heterogeneous results across studies, and this may reflect the variability in the magnetic resonance (MR) acquisition and analysis approaches across different studies and institutions, as well as MR vendors (5–7). Given the potential for DCE-MRI imaging metrics to provide early indicators of therapy-induced changes in the tumor microenvironment, it is imperative to obtain a better understanding of these imaging biomarkers for guiding adaptive and potentially individualize therapy approaches in the future. A recent 4-dimensional temporal dynamic analysis (TDA) method, which enables voxel-based, parametric analysis based on patient-specific dynamic behavior of contrast flow, may provide a more standardized approach for DCE-MRI analysis, including its validation against DCE-computed tomography (CT) (8). DCE-CT is a gold standard based on its high spatial and temporal resolution acquisition, and its highly linear and accurate relation between signal and contrast agent concentrate. However, reproducibility of either DCE-CT or DCE-MRI alone has been low (9, 10), and output parameters from either of the imaging techniques have not correlated well, with very few publications reporting on direct in vivo comparison in the same tumor using both imaging techniques. One preclinical paper studied reproducibility and absolute values of DCE-MRI and DCE-CT biomarkers in a C6 glioma model, highlighting that the techniques may have similar reproducibility but that their derived absolute parameter values are not equivalent (11). Two different kinetic models were used for the DCE-CT and DCE-MRI analyses in 2 different software applications. Korporaal et al. (12) and Kallehauge et al. (13) reported on in vivo comparisons of DCE-CT and DCE-MRI in patients with prostatic and cervical cancer, respectively. Despite voxel-based acquisitions, the analyses were reported in median values and gamma analysis used to assess spatial variation in kinetic parameters in Kallehauge et al. This study reports our early clinical experience with tumor perfusion measurements following SRS for brain metastases using both volumetric DCE-CT and DCE-MRI in the same patients supported by a common TDA framework. It is hypothesized that analyzing contrast enhancement data from both modalities in a unified and voxel-based approach will strengthen the correlations between their parametric output values. This supports the concept that low-molecular-weight contrast agents can indeed help derive tumor permeability and perfusion heterogeneity independent of the imaging modality provided the image analysis methods are standardized.

## Materials and Methods

### Patients and Treatment

Serial volumetric DCE-CT and DCE-MRI data from patients enrolled in a Research Ethics Board-approved clinical trial evaluating multiparametric imaging biomarkers of response to single-fraction radiosurgery (SRS) for brain metastases were included.

### Correlation Between DCE-CT and DCE-MRI Pharmacokinetic Parameters

To clinically validate the voxel-based TDA algorithm on DCE-MRI data against the DCE-CT data, the functional parametric maps of K_trans_ and area under the enhancement curve (iAUC_90_) from TDA DCE-CT and DCE-MRI were compared for their capabilities to evaluate tumor perfusion and permeability in this subset of patients with metastatic brain cancer for a total of 40 cases, that is, 14 tumors from 9 patients scanned at 3 time points. The following 3-step validation approach was taken:

(1) Evaluate the stability of arterial input function (AIF) versus vascular input function (VIF) measurements with volumetric DCE-CT and its impact on resulting CT perfusion parameters to create a gold standard benchmark.
(2) Pearson correlation (regression analysis) and Bland–Altman analyses were completed to evaluate relationships between perfusion parameters obtained from DCE-CT and DCE-MR and identify any systematic bias between the 2 imaging modalities. This was done for tumor functional histograms and for direct voxel-to-voxel comparison within the target volume.
(3) Evaluate the effect of using individualized voxel-based *T*_10_ measurements versus a fixed TR on resulting DCE-MRI perfusion metrics and compare both with DCE-CT.

### Volumetric DCE-CT Image Acquisition

DCE-CT data were acquired on a 320-section CT scanner (Aquilion ONE™, Toshiba, Japan) that has previously been extensively characterized for its use in radiation oncology as a radiotherapy simulator (14, 15) and DCE measurements (16). Scan parameters were as follows: 80 kV, 100 mA, 1-second rotation, and 0.468- × 0.468- × 1-mm reconstruction resolution. A total of 60 mL of iodixanol (Visipaque® 320) was injected intravenously at 5 mL/s synchronized with the start of scanning. The brain tumor DCE time sequence consisted of different sampling frequencies as follows: every 1.5 second for the first 30 seconds, every 5 second for the next 90 seconds, and every 10 second up to 180 seconds to allow for permeability modeling while balancing the scan dose with the measurement sampling rate. A noncontrast volume was acquired before contrast injection for baseline corrections and image registration. The associated volumetric dose index (CTDI-vol) was ∼100 mGy compared with 60 mGy typically reported for a routine adult head scan in this scan mode (17).

### DCE-MRI Acquisition

On the same day as the DCE-CT, each patient underwent MRI on a 3 Tesla Verio system (Siemens Medical Systems, Erlangen, Germany) with VQ gradients (peak amplitude, 40 mT/m; peak slew rate, 200 T/m/s) and a 12-channel head coil. MR scanning included endogenous *T*_1_ mapping using the variable flip angle (VFA) technique (18), DCE-MRI, *T*_2_-weighted fluid-attenuated inversion recovery (FLAIR) imaging (turbo spin echo acquisition with TR/echo time [TE] = 7253/96 milliseconds; field of view [FOV] = 220 × 220 mm; matrix = 320 × 320; in-plane resolution = 0.7 mm; section thickness = 3 mm, sections = 50; and scan time = 4 minutes and 8 seconds), and contrast-enhanced *T*_1_-weighted imaging (3-dimensional [3D] magnetization-prepared rapid gradient-echo [MP-RAGE], with TR/TE = 1400/2.2 milliseconds; FOV = 200 × 200 mm; matrix size = 320 × 320; in-plane resolution = 0.6 mm; section thickness = 1.5 mm; sections = 50; and scan time = 6 minutes and 25 seconds). VFA and DCE-MRI acquisitions used a 3D-fast low angle shot (FLASH) pulse sequence with the following common parameters: TR/TE = 4.8/1.86 millisecond; FOV = 220 × 200 mm; matrix = 174 × 192 × 40; in-plane resolution = 1.1 mm; and section thickness = 1.5 mm). VFA used flip angles of 2, 10, 20, and 30°, and the scan time was 50 second per flip angle. For DCE-MRI, the temporal resolution was 5.8 seconds, and 45 repetitions were acquired (scan time = 4 minute and 19 seconds). A weight-based bolus of gadolinium (Gd) contrast (Magnevist, Bayer AG, Leverkusen, Germany) was injected intravenously at 4 mL/s after a 20-s delay from the start of scanning followed by a 20-mL saline injection.

### Common Parametric Perfusion Analysis Framework

#### Image Registration.

Tumor regions of interest for each time point were delineated using semiautomated segmentation on the *T*_1_-weighted Gd-enhanced MR image by an expert observer and registered to the baseline DCE-MRI and DCE-CT images in GammaPlan v9 (Elekta, Sweden). Patients undergoing DCE-CT were imaged using a thermoplastic S-frame immobilization mask (QFix); thus, motion was not an issue. Immobilization could not be used in MRI, and varying degrees of motion were observed during the 3D-FLASH acquisitions, both across DCE-MRI frames and between the VFA images used for endogenous *T*_1_ mapping. Compensatory image registration was performed with a custom script in MATLAB (The MathWorks Inc., Natick, Massachusetts), to register all DCE-MRI images to the baseline image and all VFA scans to the 20° scan. Although every voxel in the brain scan was analyzed, clinical treatment contours were exported from GammaPlan so that the analysis could be correlated to the corresponding radiation clinical target volume.

### Signal-to-Contrast Concentration Conversion

For DCE-CT data, the CT numbers were converted from Hounsfield units (HU) to contrast concentration based on previous calibration phantom experiments with static and dynamic concentrations of contrast, resulting in a linear scaling of 33 HU/mgI/mL (19).

The DCE-MRI signal modeling used the standard equations for 3D-FLASH magnitude signal conversion to Gd-diethylenetriaminepentaacetic acid (DTPA) concentration as follows:

(1) $$S=S_{0}\sin\alpha \frac{\left(1-E_{1}\right)}{\left(1-E_{1}\cos\alpha \right)}e^{\frac{-T_{E}}{T_{2}^{*}}}$$

where *E*_1_ = exp(−TR/T_1_), ⁡ is the flip angle, and *S*_0_ and *S* are the relative signal enhancements before and after contrast injection, respectively (20).

Magnitude signal enhancement was converted to signal using the following equation:

(2) $$\frac{1}{T_{1}}=\frac{1}{T_{10}}+r_{1}C,$$

where *T*_10_ and *T*_1_ are the spin-lattice TRs before and after contrast injection, respectively, *r*_1_ is the relaxivity of the contrast agent at 3 T (3.3 L/mmol/s) (21), and C is the concentration of Magnevist. For dynamic image analysis, the average of signals at the first 3 time points provided an estimate of the signal baseline. The voxel-based *T*_10_ maps were the VFA *T*_1_ maps derived for every patient and every imaging session using precontrast signal profiles measured at 2, 10, 20, and 30° flip angles (18).

### Voxel-Based Pharmacokinetic Modeling

The TDA algorithm applies a classification scheme to each voxel on the basis of temporal characteristics of the voxel's contrast enhancement over time and then creates parametric maps within these TDA-derived masks based on a specified kinetic model to iteratively improve classification and parameter sensitivity (22). The modified Tofts model (23) is commonly used in patients with brain perfusion on the basis of the hypothesis of weak vascularization and increased permeability in tumors (24, 25). It describes the arterial input, C_a_(t), and tissue enhancement curve, C_t_(t), as follows:

$$C_{t}(t)=\frac{K_{trans}}{1-Hct}(C_{a}(t)⊗e^{-k_{ep}(t-z)}+V_{b}C_{a}(t)$$

In addition to semiquantitative measures as the integrated iAUC_90_, the following are the resulting functional parameters of interest: K_trans_, the transfer constant from the blood plasma into the extracellular extravascular space (EES); K_ep_, the transfer constant from the EES back to the blood plasma; and v_e_, the extravascular extracellular volume. V_b_ is the whole blood volume per unit of tissue (mL/g). The hematocrit value, H_ct_, was assumed to be 0.4 for all cases. The AIF_CT_ was chosen in the internal carotid artery for DCE-CT and compared with VIF_CT_ in the sagittal sinus. For this study, a population-based input function (AIF_MRI_) was used for DCE-MRI analysis because of variability in the flip angle between patients (26) and it allowed for a robust comparison of the impact of the analysis methodology against DCE-CT (27). A 3D voxel mask, as well as a separate sum of squared errors mask, was created for each functional parameter to show the quality of fit of the transport model. Finally, a histogram moment analysis was performed for each parameter inside the tumor mask for assessing the standard deviation, skew, and kurtosis of the histogram shape.

### Statistical Analysis

Statistical analysis was performed in Matlab. The histogram parameters estimated by volumetric DCE-CT and DCE-MRI were compared via Bland–Altman analysis, in which differences in perfusion parameter values between the modalities were plotted against the mean of the pair of values, and Student *t* test and Pearson correlation. All statistical analyses were 2-sided and values with *P* < .05 were deemed statistically significant. Direct voxel-to-voxel comparison was done in a similar way with the addition of a variance component analysis to estimate inter-day variance (28).

## Results

### Patient Demographics

A cohort of 9 patients with a total of 14 metastatic brain tumors (lung cancer, 3; breast cancer, 3; melanoma, 2; and squamous cell carcinoma, 1) underwent imaging at baseline (day 0—before radiosurgery), day 7, and day 20 after radiosurgery (total number of data sets = 40). One patient with 2 tumors missed the day 7 appointment. SRS with a mean dose of 20.5 Gy (18–21 Gy) using GammaKnife Perfexion (Elekta, Sweden) was performed.

### VIF Selection

**Figure 1.**
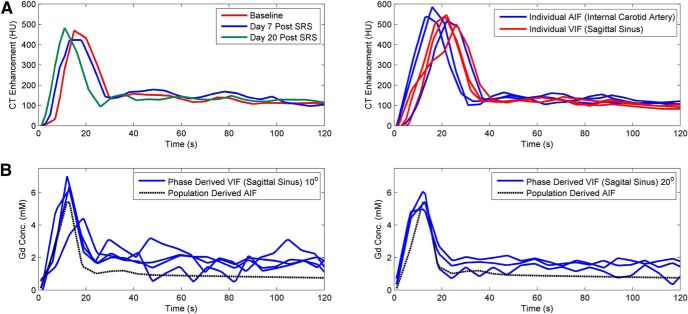
Example arterial input functions (AFIs) from dynamic contrast-enhanced computed tomography (DCE-CT) over different imaging days and comparison of AIF and vascular input function (VIF) for 1 patient (A). Example phase-derived VIF at a flip angle of 10° (left) and 20° (right) compared with a population-based AIF (B).

**Figure 2.**
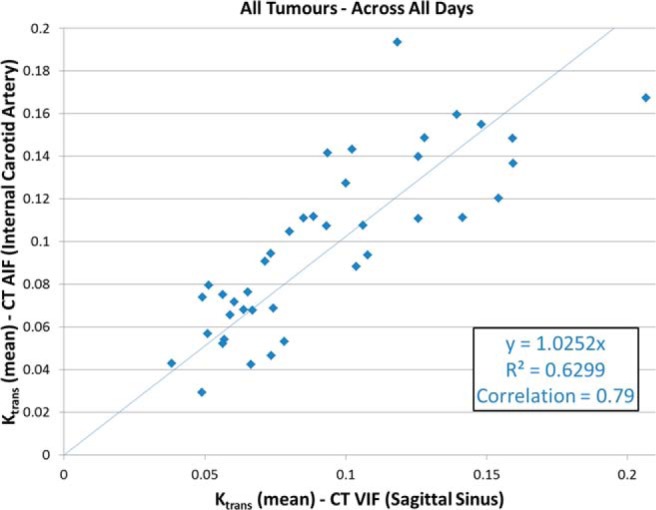
Correlation between mean K_trans_ values from DCE-CT using AIF from the internal carotid artery versus a VIF from the sagittal sinus, over all tumors and imaging days.

VIFs for voxel-based kinetic analysis were selected in the internal carotid artery (AIF_CT_) and sagittal sinus (VIF_CT_). The AIF_CT_ curves for patient 1 are shown in Figure 1A, together with the population-based AIF_MRI_ curve, whereas Figure 1B highlights the (small) variations in input curves for all DCE-CT measurements. The mean AIF_CT_ peak (438 ± 68 HU) was slightly higher than VIF_CT_ (382 ± 100 HU), with the corresponding AIF_CT_ onset time (6.7 ± 3.5 seconds) earlier than VIF_CT_ (12.2 ± 4.1 seconds). The bottom panels of Figure 1 show examples of individual-phase MRI measurements of AIF for 2 different flip angles. Given the variation in flip angle acquisition during the clinical trial, it was decided to use a population-based AIF for the DCE-MRI analysis since they are quite similar to the CT-based AIF. The impact of using AIF_CT_ or VIF_CT_ as the input function for parametric modeling is shown to provide equivalent performance in Figure 2, indicating the high correlation and interchangeability between the uses of either input function.

### Tumor Perfusion Evaluation: Volumetric TDA CT vs MRI

#### Median Histogram Correlation.

Statistically significant moderate correlations between MR and CT were found for median K_trans_ (R = 0.42, *P* = .01) and median iAUC_90_ (R = 0.40, *P* = .01) over all time points (n = 40; 1 patient was removed because of blooming leakage effect seen on MRI). The variation over the different imaging days is listed in more detail in Table 1 with a strong correlation at baseline for median K_trans_ (R = 0.513, *P* = .008) and median v_e_ (R = 0.58, *P* = .03). The percentage change in median K_trans_ and v_e_ over time showed a statistically significant correlation for early (day 7) change relative to that for the baseline (R = 0.64, *P* = .02) but not at day 20 (R = 0.07, *P* = .81), which is likely related to the small brain metastases volumes at day 20 (mean of volumes at day 20 = 1.4 cc). The percentage change in median iAUC_90_ values (−25% ± 67%) did not significantly correlate on either day 7 (*P* = .29) or day 20 (*P* = .94).

**Table 1.** Statistically Significant Correlations and Variations

Statistical Correlations						
Median	All Days	Day 0	Day 7	Day 20	Early (Day 7)	Day 20
		**K_trans_**			**%Change K_trans_ from Baseline**	
R	0.421	0.513	0.213	0.492	0.641	−0.111
P	0.01	0.008	0.505	0.074	0.025	0.707
Bias	0.051	0.058	0.045	0.05	0.072	0.042
LoA	0.105	0.085	0.131	0.106	1.012	1.901
		**iAUC_90_**			**%Change iAUC_90_ from Baseline**	
R	0.404	0.216	0.641	0.277	−0.062	−0.299
P	0.01	0.457	0.025	0.338	0.848	0.3
Bias	0.025	0.036	0.02	0.018	−0.069	−0.112
LoA	0.103	0.105	0.079	0.121	1.173	1.15
		**v_e_**			**%Change v_e_ from Baseline**	
R	0.164	0.587	0.414	0.203	0.453	0.127
P	0.313	0.027	0.181	0.487	0.139	0.665
Bias	0.339	0.028	0.083	0.869	0.081	2.268
LoA	0.817	0.143	0.634	0.644	1.612	19.474

(A)						
Histogram Analysis	K_trans_			iAUC_90_		
	Median	Skew	Kurtosis	Median	Skew	Kurtosis
Day 0						
R	0.51	−0.09	0.02	0.60	0.26	0.25
P	0.05	0.75	0.95	0.02	0.35	0.36
Day 7						
R	0.21	0.05	0.07	0.70	0.30	0.16
P	0.51	0.89	0.83	0.01	0.35	0.62
Day 20						
R	0.49	0.15	0.01	0.37	0.44	−0.02
P	0.06	0.60	0.98	0.18	0.10	0.93
Combined						
R	0.42	0.06	0.03	0.55	0.34	0.15
P	0.01	0.69	0.85	0.00	0.03	0.34
**(B)**						

This table present Pearson correlations, significance, and Bland–Altman values between (**A**) median parametric values derived from DCE-CT compared with those derived from DCE MRI and (**B**) other histogram metrics for K_trans_ and AUC parameters. K_trans_, the transfer constant from the blood plasma into the EES; v_e_, the EES volume; and iAUC_90_, the integrated area under 90% of the curve. Statistically significant values are underlined.

**Figure 3.**
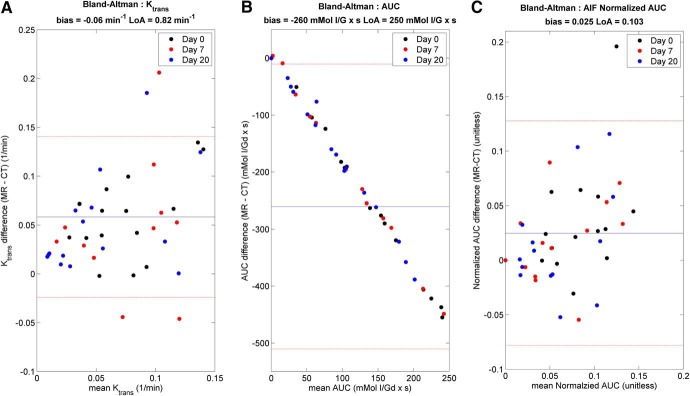
Bland–Altman plots comparing DCE-CT and DCE-magnetic resonance imaging (MRI) for measurement of perfusion parameters: median Kt_rans_ (A), median iAUC_90_ (B) and normalized iAUC_90_ (C). Mean differences are denoted as solid blue lines, and the 95% confidence limits are denoted as dashed red lines.

Bland–Altman agreement between volumetric DCE-CT and DCE-MRI is shown in Figure 3. K_trans_ median (Figure 3A) and iAUC_90_ median (Figure 3B) values remained largely within 0.105 min^−1^ Limits of Agreement (LoA) with some bias (0.05 min^−1^), suggesting that the 2 modalities may be interchangeable. The absolute K_trans_ values from DCE-CT are slightly lower than those from DCE-MRI. A strong correlation between iAUC_90_ values from CT and MRI is shown in Figure 3B, which exhibits a linear trend, as the bolus volumes of Visipaque® and Gd-DTPA contrast agent injected were different but consistent, and because the iAUC_90_ parameter is a cumulative measurement of enhancement, it appears as a proportional bias. This bias disappears when normalizing to the bolus amount injected, and the normalized iAUC_90_ shows little bias (0.025) and small LoA (0.103) values (Figure 3C). In terms of other histogram metrics, only the skewness change in iAUC_90_ has any significant correlation between the 2 modalities (*P* = .03).

### Voxel-Wise Correlation

**Figure 4.**
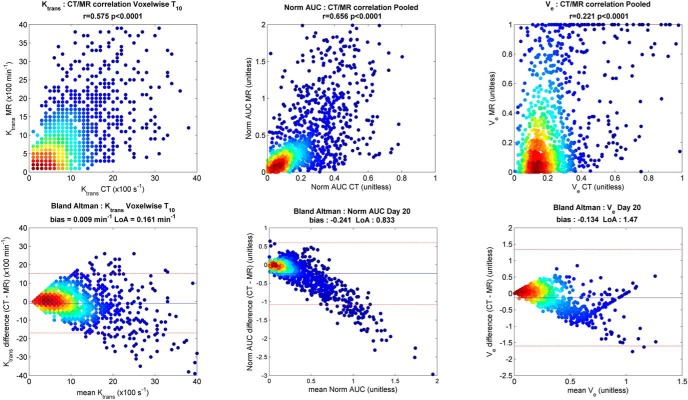
Comparison of voxel-wise K_trans_ measurement from volumetric DCE-CT and DCE-MRI over all imaging days for 1 patient: Pearson correlation plot (Left); Bland–Altman plot illustrating technique interchangeability with the mean difference denoted as a solid blue line and the 95% limits of agreement denoted as dashed lines (Right).

Modalities were also compared on a direct voxel-to-voxel basis within the tumor regions of interest. With a resolution of 128 × 128 × 40 matched to both CT and MRI, moderate-to-strong correlations in K_trans_ values were found for day 0 (R = 0.37, *P* < .001), day 7 (R = 0.74, *P* < .001), and day 20 (R = 0.52, *P* < .001). The correlation over all imaging days combined is shown in Figure 4. The color gradient highlights the density (frequency) of K_trans_ values from low incidence (blue) to high incidence (red). The Bland–Altman plot shows excellent agreement for K_trans_ and normalized area under the curve values between modalities over all days, with very little evidence of K_trans_ bias (0.009 min^−1^, LoA 0.16 min^−1^). Correlation of voxel-wise v_e_ was statistically significant but relatively low with an R value of 0.22.

### Sensitivity of DCE-MRI Perfusion Modeling Parameters to Individualized *T*_10_ Values

**Figure 5.**
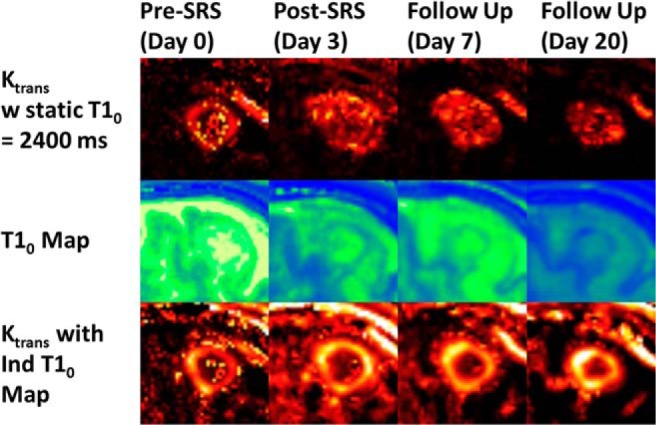
Central section through the tumor for the same patient over the different imaging days showing (top) K_trans_ values using a static *T*_10_ map, (middle) the voxel-based *T*_10_ map, and (bottom) K_trans_ values using the individual *T*_10_ map.

**Figure 6.**
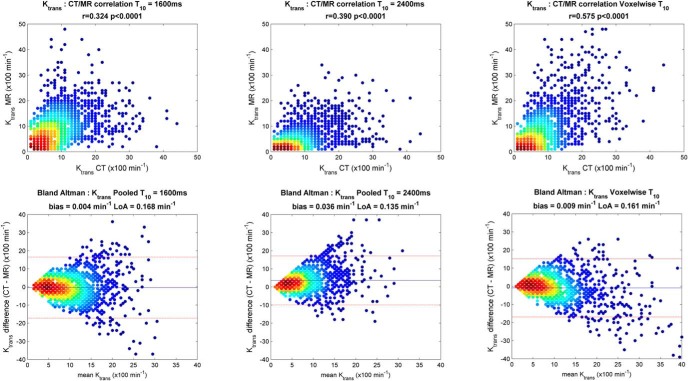
Comparison of voxel-wise K_trans_ measurement from DCE-CT to DCE-MRI using *T*_10_ value of 1600 milliseconds (Left) versus 2400 milliseconds (Middle) and individual *T*_10_ from VFA *T*_1_ measurement (Right).

It was hypothesized that individual precontrast TRs' *T*_10_ values would make a significant difference in resulting perfusion parameters as per a prior study by Heye et al. (6) in patients with cervical cancer. Figure 5 shows the qualitative impact of using a global *T*_10_ of 1600 milliseconds or 2400 milliseconds versus individual voxel-based *T*_10_ maps on K_trans_ values for 1 patient at the different imaging days (29). The higher *T*_10_ value was based on experimentally probing the highest voxel-based values in a number of tumors. The lower value is based on the measured median tumor *T*_10_ value over all available data, which was 1572 ± 594 milliseconds (n = 41). Using a constant *T*_10_ value of 2400 milliseconds over voxel-wise *T*_10_ resulted in significantly higher K_trans_ (0.3 ± 0.14 min^−1^) and iAUC_90_ values (*P* < .0006) compared with CT. Use of a static *T*_10_ value of 1600 milliseconds produced a regression correlation between CT and MRI K_trans_ values that was closer to the voxel-based *T*_10_ results. This is reflected in Figure 6 which shows the highest voxel-wise correlation between K_trans_ values from CT and MRI_*T*_10__ (R = 0.575, *P* < .0001) for all imaging days and patients, including good interchangeability as can be seen in the Bland–Altman plot.

## Discussion

This work investigated the use of a unified analysis platform (based on the TDA implementation) to compare DCE-MRI against DCE-CT parameters in patients with brain metastases treated with SRS. The use of volumetric DCE-CT was considered a gold standard given its linear signal-to-contrast concentration relationship and proven robustness (22).

Based on DCE-CT data, AIF and VIF appear to be interchangeable in generating similar K_trans_ values. This confirms that the use of individual VIF in DCE MRI analysis is a reasonable approach. In contrast, the application of different *T*_10_ values considerably impacted the resulting K_trans_ values (Figures 5 and 6), as suggested by Heye et al. (6). The use of individual *T*_1_ mapping with voxel-wise precontrast TRs for each DCE-MRI image set in the pharmacokinetic analysis resulted in the highest voxel-wise correlation between K_trans_ values from CT and MRI_*T*_10__ (R = 0.575, *P* < .0001) across all imaging days and in all patients. This approach is likely to provide more accurate quantitative evaluation of parametric tumor heterogeneity (6).

Our results show that the use of the TDA approach for both DCE-MRI and DCE-CT data results in well-correlated (R = ∼0.5) median DCE parameter values in the tumor (K_trans_, iAUC_90_, and V_e_). This correlation increased even more (R = 0.77) when performing a direct voxel-wise analysis (K_trans_ and iAUC_90_) and, in doing so, capturing tumor heterogeneity. The voxel-wise correlation of extravascular volume fraction, v_e_, was low between the 2 modalities, but it was highly statistically significant (R = 0.22, *P* < .001). Because this parameter is highly dependent on K_ep_, the transfer constant from the EES goes back to the blood plasma; this discrepancy can be explained by the differences in the molecular weight and the composition of the 2 contrast agents affecting their diffusion and extraction fraction in the interstitial space. In the evaluation of median 3D tumor volume histogram, the Bland–Altman plots showed significant interchangeability, but there was some bias (0.05 min^−1^) toward higher MRI perfusion values. Using the 0.02 absolute differences in K_trans_ values between the AIF_CT_ and VIF_CT_ correlation as a standard error, this offset may or may not be statistically relevant.

This bias disappeared with voxel-wise Bland–Altman analysis, which suggests that the 2 modalities may be interchangeable when assessing the vascular permeability of brain metastases in a voxel-based approach. Other histogram values such as the skew or kurtosis of the parametric distributions were not statistically significant. This further suggests that voxel-based analysis is required to capture tumor heterogeneity and that this is not necessarily a normal distribution.

The correlations of DCE parameters were consistently lower at day 20 than at day 7 for both absolute values and their relative change from baseline. This is likely due to the very small tumor volumes (1.4 cc, range: 0.1–5.3 cc) at a later time point, which, consequently, will result in a lower number of voxels available for reliable correlative statistics.

The correlations found in this work are significantly higher than those previously reported using different analysis methods with nonvolumetric DCE-CT measurements and/or limited image registration between the different modalities (30). Kallehauge et al. (2013) reported stronger correlation values when comparing DCE-CT and DCE-MRI in locally advanced cervical cancer (13) using a gamma similarity measure and scaling the DEC-CT results based on the amount of injected contrast compared with DCE-MRI.

At the time this imaging study protocol was developed and the trial was started, the QIBA profile for DCE-MRI recommended a VFA technique for *T*_1_ mapping. As the study had started with the VFA technique, the same technique was continued for the duration of the study.

Other *T*_1_ mapping techniques—such as the use of inversion pulses or incorporation of time-efficient RF mapping—may have greater accuracy and could be explored for future studies.

Some of the remaining differences may be inherent to the type and molecular weight of the injected contrast agent, with iodexol (CT) being larger and heavier than Gd-DTPA (MRI). This is expected to affect its transport capabilities across the same capillary network and could explain the lower correlation in v_e_ fraction.

In summary, our results validated the use of the TDA method as a common analysis approach for both DCE-MRI and DCE-CT data through strong voxel-wise histogram correlation across modalities and highlighted the need for voxel-wise, individualized *T*_10_ mapping in patients to derive meaningful DCE metrics.

**Advances in Knowledge**

(1) High correlation between DCE-MRI and DCE-CT using TDA validates the use of this common platform for both quantitative and semiquantitative parameters across imaging modalities, which will enable standardized functional analysis methods.
(2) Voxel-wise histogram analysis of perfusion and permeability values better elicits tumor heterogeneity and results in significantly higher correlations between modalities compared with region of interest-based (mean/median) values.
(3) The assumed value for *T*_10_ precontrast relaxation significantly impacts the accuracy of heterogeneous pharmacokinetic maps, and the strongest correlation between DCE-MRI and DCE-CT was observed when individually measured voxel-wise *T*_10_ maps were implemented.
(4) Volumetric DCE-CT analysis showed that the input to pharmacokinetic calculations in the CT could be an AIF or VIF measurement, as the resulting parameters showed significant agreement with little to no bias.

**Implications for Patient Care**

(1) The implementation of individually measured voxel-wise precontrast relaxation maps is strongly recommended when quantitative pharmacokinetic analysis with DCE-MRI is planned given its impact on the resulting parameter accuracy.
